# Supplementary material for: Complexome profiling of the Chlamydomonas psb28 mutant reveals TEF5 as an early PSII assembly factor
Source: Plant Cell. 2025 Mar 17;37(6):koaf055. doi: 10.1093/plcell/koaf055 (PMC12164590; doi:10.1093/plcell/koaf055)
Supplement: koaf055_Supplementary_Data [file koaf055_supplementary_data.zip › psb28_interactive.zip!/plots/intensity/qualityPlots/peptides/IDH2_Cre02.g143250.t1.2.html]

### Cre02.g143250.t1.2

Trivial name: IDH2  
SeqCoverage [median]: 51.00  
Mass: 36.84 kDa  
  
 

| Sequence | MissedCleavage | Mass [Da] | wtIntensity | muIntensity | Score |
| --- | --- | --- | --- | --- | --- |
| ANPTALLLSSAMMLR | 0 | 1587.85 | 2328.58 | 5295.01 | 81.90 |
| AVIDKLE | 1 | 786.45 | 0.00 | 10186.79 | 116.88 |
| ENLDSVLK | 0 | 916.49 | 7044.30 | 22827.57 | 107.01 |
| ENTEGEYSGLEHEVVPGVVESLK | 0 | 2500.20 | 5479.90 | 49266.62 | 114.04 |
| GPMATPIGK | 0 | 870.46 | 13076.36 | 35816.64 | 183.90 |
| IAEFAFAYAR | 0 | 1157.59 | 35028.11 | 36895.46 | 131.06 |
| IGLKGPMATPIGK | 1 | 1281.75 | 26288.16 | 27242.56 | 103.14 |
| IPVVWDEQHIGK | 0 | 1419.75 | 3173.45 | 6164.98 | 58.67 |
| NKANPTALLLSSAMMLR | 1 | 1829.99 | 2969.65 | 2905.49 | 58.09 |
| RQEGDNIQNAVLGVIAEGK | 1 | 2010.05 | 13794.35 | 7638.39 | 96.01 |
| SFEATLFPGDGIGPEIAASVR | 0 | 2133.07 | 556.91 | 0.00 | 52.81 |
| SLNLTLR | 0 | 815.49 | 7797.99 | 33433.07 | 107.90 |
| SLNLTLRK | 1 | 943.58 | 0.00 | 1791.20 | 83.27 |
| TADLGGNATTSDFTK | 0 | 1497.69 | 46697.55 | 67829.09 | 306.06 |
| TNSMVTR | 0 | 807.39 | 0.00 | 594.76 | 148.99 |
| TRYDGINLVTVR | 1 | 1405.77 | 15921.99 | 55998.93 | 77.60 |
| YDGINLVTVR | 0 | 1148.62 | 12977.24 | 37008.28 | 139.02 |
| YRTADLGGNATTSDFTK | 1 | 1816.86 | 1143.72 | 641.21 | 52.86 |
|  |  |  | 194278.2 | 401536.1 |  |
